# How Bad Is It? Usefulness of the "7eed Model" for Scoring Severity and Level of Need in Complex Emergencies

**DOI:** 10.1371/currents.dis.d59e0fa39887031e1c3763851a6e5c2a

**Published:** 2016-06-28

**Authors:** Anneli Eriksson, Martin Gerdin, Richard Garfield, Thorkild Tylleskar, Johan von Schreeb

**Affiliations:** Department of Public Health Sciences, Health System and Policy, Karolinska Institute, Stockholm, Sweden; Department of Public Health Sciences, Health System and Policy, Karolinska Institute, Stockholm, Sweden; ERRB, Centers for Disease Control, Atlanta, Georgia, USA; Centre for International Health, University of Bergen, Bergen, Norway; Department of Public Health Sciences, Health System and Policy, Karolinska Institute, Stockholm, Sweden

## Abstract

**Background:**

Humanitarian assistance is designated to save lives and alleviate suffering among people affected by disasters. In 2014, close to 25 billion USD was allocated to humanitarian assistance, more than 80% of it from governmental donors and EU institutions. Most of these funds are devoted to Complex Emergencies (CE). It is widely accepted that the needs of the affected population should be the main determinant for resource allocations of humanitarian funding. However, to date no common, systematic, and transparent system for needs-based allocations exists. In an earlier paper, an easy-to-use model, “the 7eed model”, based on readily available indicators that distinguished between levels of severity among disaster-affected countries was presented. The aim of this paper is to assess the usefulness of the 7eed model in regards to 1) data availability, 2) variations between CE effected countries and sensitivity to change over time, and 3) reliability in capturing severity and levels of need.

**Method:**

We applied the 7eed model to 25 countries with CE using data from 2013 to 2015. Data availability and indicator value variations were assessed using heat maps. To calculate a severity score and a needs score, we applied a standardised mathematical formula, based on the UTSTEIN template. We assessed the model for reliability on previous CEs with a “known” outcome in terms of excess mortality.

**Results:**

Most of the required data was available for nearly all countries and indicators, and availability increased over time. The 7eed model was able to discriminate between levels of severity and needs among countries. Comparison with historical complex disasters showed a correlation between excess mortality and severity score.

**Conclusion:**

Our study indicates that the proposed 7eed model can serve as a useful tool for setting funding levels for humanitarian assistance according to measurable levels of need. The 7eed model provides national level information but does not take into account local variations or specific contextual factors.

## Background

Humanitarian assistance is designated to save lives and alleviate suffering among people affected by disasters 1^,^^2^. In 2014, close to 25 billion USD was allocated to humanitarian assistance globally, the bulk of it from governmental donors and EU institutions 3. The majority of humanitarian funding is channeled through UN coordinated appeals to contexts that that can be defined as complex emergencies (CE) 3. Major donors have agreed that need should determine funding priorities for humanitarian assistance, and that allocations should be made in proportion to needs 2^,^^4^.

There is, however, no agreed model or tool to help determine needs-based humanitarian funding 5^,^^6^.

The process of assessing the overall severity of the situation challenge funders. The tools needed to address this challenge are different than the operational needs assessments tools that provide detailed information on needs to guide interventions 6^,^^9^ . Existing tools typically 1) look at specific sectors, 2) other are field and intervention oriented or 3) assess severity globally but do not sufficiently discriminate among the most severe CEs. Below some examples:

1) The IPC (Integrated Food Security Phase Classification) is a specific sector oriented index that looks at food security and allows a comparison between contexts but only in this area 22.

2) The PDNA (Post Disaster Needs Assessment) is a tool to define sector needs after disasters 23 . It is to a large extent a tool to be used at the disaster site. So is the MIRA manual (multi sector initial rapid assessment) to be used after sudden onset disasters. It provides guidance for remote assessments but focus lays on “at site assessments” 24.

3)Several initiatives such as the Global Emergency Overview (GEO), the Global Needs Assessment (GNA), Index for Risk Management (inFORM) and the Global Focus Model (GFM) have in recent years been developed to provide information on global needs and risks. However, where these initiatives define needs, they do not distinguish between levels of needs among the worst CE affected countries and do not allow for comparison of severity and magnitude of needs 8^,^^9^^,^^10^^,^^11^.

To enable needs-based humanitarian funding we developed a model using quantifiable indicators to measure and compare severity of and between CEs [13], the “7eed model”, a combination of both severity and need - seve(rity)need. The 7eed model is presented in Annex 1. The aim of this study was to determine the usefulness of the model by assessing 1) availability of the indicator data, 2) variations between CE effected countries and sensitivity to change over time, and 3) reliability in capturing severity.

## Setting

Our 7eed model was developed for Complex Emergencies (CE), defined as a situation with a breakdown of authority due to conflict that requires a multi-agency response 13. In our definition we also included situations where the mortality among the civilian population has increased significantly compared to baseline, due to direct or indirect causes of conflict, such as malnutrition and/or spread of communicable diseases 14. We also include contexts where governmental policies contribute to the development of a disastrous situation, such as food insecurity and high rates of malnutrition, or so-called complex political emergencies 14^,^^15^. We defined reliability as the agreement between the model output and severity, and we defined severity as excess mortality.

**The 7eed Model**

The Utstein framework 12 is:“A conceptual framework that describes the progression of a hazard that becomes an event, which causes structural damage and a decrease or loss of function (functional damage), that, in turn, produces needs that lead to a disaster” 16.

In our 7eed model we kept the Utstein logic, but adapted it to situations where the hazard is an on-going event. The model has the following components:

**Disaster severity = Vulnerability X Exposure**

As a next step – to relate severity with level of need - we suggested that the number of people in need is used as a single factor in the model in the following way:

**Level of Need = Severity X People in Need**

How we identified and selected the indicators of vulnerability and exposure included in the model has been described previously 11.The 7eed model is presented in Annex 1.

The 7eed model provides an estimate of the severity of a CE based on the resulting score of 4 indicator values for vulnerability and 4 for exposure. The vulnerability indicators were assigned scores according to the 2012 Human Development Index values for the least developed countries. The exposure indicators were assigned scores using information from the UN-consolidated appeals for 2012.

## Method

To test the 7eed model, we conducted the following analyses:

1. We applied the 7eed model to 16 countries affected by CE over three consecutive years. Nine additional countries were assessed for one or two years. With these results, heat maps were developed and reviewed for indicator availability, variations over time, and variations between countries.

The 16 countries selected for inclusion were the ones that had UN consolidated appeals assembled during this period. They were therefore deemed relevant for severity scoring and funding decisions. We used the definition for CE presented above to identify these countries. The countries were: Afghanistan, Burkina Faso, The Central African Republic (CAR), Chad, Djibouti, Democratic Republic of Congo (DRC), Haiti, Mali, Mauretania, Niger, occupied Palestinian Territories (OPt), Somalia, South Sudan, Sudan, The Syrian Republic and Yemen. In 2013 and 2014, Kenya and Zimbabwe were selected, and in 2015 Cameroon, Gambia, Iraq, Myanmar, Nigeria, Senegal and Ukraine were added.

Most of these countries are or were at the time going through periods of armed conflict or instability. In the Sahel region food insecurity and high levels of malnutrition were the driving causes of the disaster, aggravated by displacement and regional or internal instability. For Zimbabwe food insecurity was the main cause of the disaster and for Kenya and Djibouti food insecurity and high numbers of refugees were the main cause.

We used the World Banks data web (http://data.worldbank.org) to obtain values for the vulnerability indicators. When information was unavailable or the values were older than three years, we searched for information from other sources, such as Multiple Indicator Cluster survey (MICS) and other surveys referred to in the UN appeals. If no indicator value was found, we marked the indicator information as not available (na).

Values for the exposure indicators were derived from UN appeals. We obtained data for 2013 and 2014 from CAP documents, and for 2015 from the UN’s Humanitarian Response Plans. In 2015, the UN appeals changed the terminology from “affected” to “people in need of humanitarian assistance” which is estimated using a coordinated field process where data on humanitarian needs are consolidated 17.

We subsequently changed the terminology in our model to “people in need”, as the terms are comparable. Values on “number of uprooted” was from UNHCR, if unavailable in the appeal. To obtain information on the proportion data, total population size was obtained from the UN appeals or the World Bank database. The framework was populated with indicator values and scored according to predefined cut-offs for each individual country.

The results were presented as heat maps using colour coding to determine the different levels of severity for the indicator values; yellow indicates low/medium severity, orange is high severity, and red is extreme severity. When information or values were unavailable, the square was left blank.

2. We established a standardised mathematical formula, building on the UTSTEIN framework to calculate a severity and needs score

During scoring, each indicator was given a numeric value based on the indicator severity from 0,5 (low-medium) to 1 (high), to 1,5 (extreme). Vulnerability and exposure were added up separately and then multiplied. The severity score could thus range from 4 (lowest severity) to 36 (highest severity). To obtain a needs score, the number of people affected or in need was added to the equation by multiplying by the number of millions affected (2013 and 2014) or in need (2015).

3. We tested the 7eed model for reliability on a number of previous complex disasters with a documented outcome of excess mortality.

Excess mortality is a recognized measurement of severity of a disaster situation 18. However, excess mortality is a late sign of a deteriorating situation and mortality data is often difficult and complex to obtain 19^,^^20^. In CE, excess mortality is particularly challenging to determine. Consequently there is lack of reliable data. Following a careful search of available publications, we decided to use information on excess mortality in CEs from a publication by The Geneva Declaration Organization 21. This is a referenced publication on the subject and attempts to methodically assemble and compare data on mortality in conflict. Estimates of direct and indirect mortality per 100 000 people, per year were used to assess the 7eed model, in the following way: Information on mortality from 11 conflict-related complex emergencies that occurred partly or completely between 1993 and 2005 was used. The countries were Kosovo (1998–1999), Iraq (2003–2007), Northern Uganda (2005), Democratic Republic of the Congo (DRC) (1998–2002), Burundi (1993–2003), Sierra Leone (1991–2002), Darfur, Sudan (2003–2005), South Sudan (1999–2005), Angola (1975–2002), Liberia (1989–1996), and East Timor (1974–1999).

We applied the 7eed model on the same countries with values for the different indicators from the relevant years and compared the severity score of the countries with excess mortality rates.

## Results

**Availability of indicator data:** All the vulnerability indicator information was available for eleven of the sixteen countries assessed over three years. Five countries had missing data in 2013, but by 2015 only Somalia lacked information, and only on one vulnerability indicator. The indicators adult literacy and underweight were updated less frequently than once per year. Indicators for exposure were included in the majority of the UN appeals. For the number of uprooted people, the UNHCR website also provided annually updated information, while the UN appeals updated this information for every update in the appeal, depending on the situation. Information on number of people affected by the CE was not consistently presented; sometimes it was presented as per intervention sector, such as health or food security, and sometimes presented as an overall number. When presented per sector, the total number of affected was uncertain, as a significant overlap between sectors was likely. For countries where the number of affected per sector varied significantly (Afghanistan, Burkina Faso, Mauretania, Zimbabwe), we calculated a median number. The number of people in need was available for the all assessed countries in the 2015 UN appeals.

**Variations between countries and sensitivity to change over time: **The vulnerability scoring showed variations among countries. No country scored red on all four vulnerability indicators (extreme); however, Somalia scored red on the three available indicators. For 2015, four countries had an overall yellow score (low – medium). These were Iraq, oPT, Syria and Ukraine. Seven of the 16 countries assessed over three years had vulnerability indicators that improved over time.

The exposure indicators had significant variations among the countries, with four countries standing out with red for three out of four scores (extreme) for at least two of the three years. These were oPT, Somalia, South Sudan, Sudan and Syria. For oPT, the number and proportion of uprooted includes a majority of people uprooted several decades previously. None of the countries had an all yellow score for exposure over three years, although Haiti, Mauretania, and Ukraine had an all yellow score for exposure in January 2015.

**Heat maps 2013 – 2015 for 16 CE affected countries with Consolidated UN appeals:**
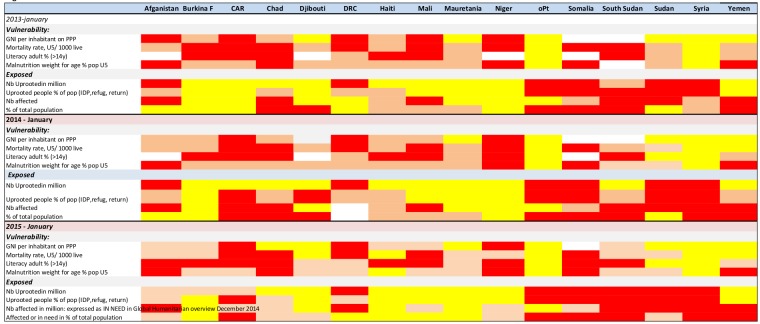

**Heat maps for additional CE countries and countries linked Sahel countries with on-going food insecurity 2015:**
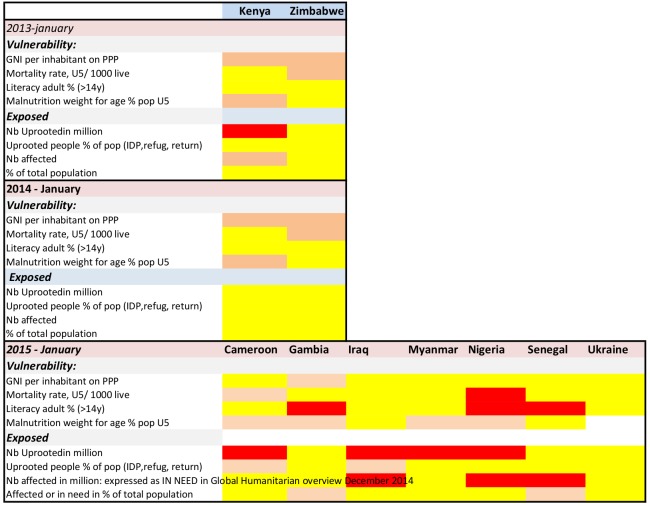

**Variations between countries - scoring of severity and needs.**

**Severity score January 2015:**
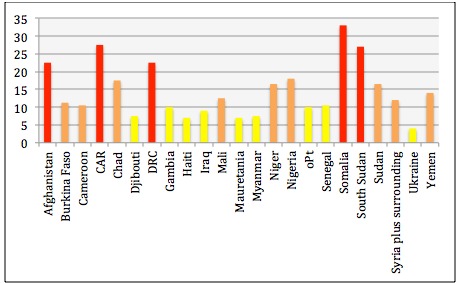
The Y-axis displays the severity score based on vulnerability X severity, with a possible variation between 4 and 36. In the graph a severity score above 20 results in a red bar, severity between 10 and 19 an orange bar and severity below 10 a yellow bar.

The severity score shows the variation between countries. While the possible severity scores ranged from 4 and 36, the highest score in the assessment was 33, which was assigned to Somalia. For Somalia, information on one of the vulnerability indicator GNI per inhabitant on PPP was missing. In the calculation we assumed that the value of the indicator would be extreme, based on our understanding of the context.

The scores ranged from 4 to 33. In total, five countries scored above 20: Afghanistan, CAR, DRC, Somalia and South Sudan. Ukraine had the lowest score of 4, as both vulnerability and people displaced and in need were relatively low.

**Scoring of needs** - for assessed countries in 2015 is presented in figure 4.

**Need score January 2015:**
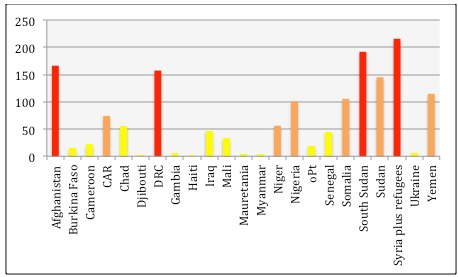
The Y-axis displays the need score based on severity score X million in need. In the graph a need score above 150 results in a red bar, a need score between 50 and 149 an orange bar and a need score below 50 a yellow bar.

The need score had wide variation among countries. The highest score was 216 for Syria and the region, while the lowest was below 2 for Djibouti. Afghanistan, DRC and South Sudan all had high severity and high need scores. CAR and Somalia had the highest severity scores but lower need scores, while Syria and the region had the absolute highest need score, but only the tenth highest severity score.

**Previous CE – assessing the reliability of the model in capturing severity.**

**Heat map, severity score and excess mortality for 11 historical CEs:**
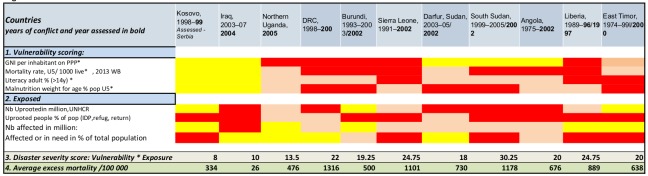

In the table, a heat map for the eleven countries is presented and below the severity score and the estimated excess mortality per 100 000 people. In figure 6 the severity score and excess mortality is plotted.

**Plotting of severity score and average excess mortality/year:**
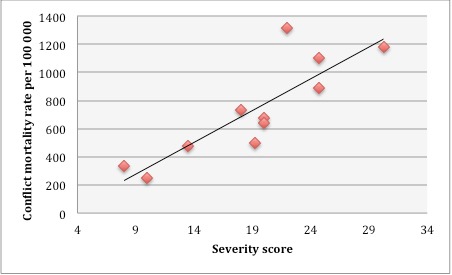
Countries plotted: Angola, 1975–2002, Burundi, 1993–2003, Darfur, Sudan, 2003–05, East Timor, 1974–99, Northern Uganda, 2005, DRC, 1998–2002, Liberia, 1989–96, Sierra Leone, 1991–2002, South Sudan, 1999–2005, South Sudan, 1999–2005, Iraq, 2003–07, Kosovo, 1998–99

The severity score follows the estimated excess mortality in ten of the eleven tested historical CE countries. The exception is DRC, where the estimated excess mortality suggests a more severe situation than what we found when we applied the severity scoring model to the same context

## Discussion

Our assessments indicate that the 7eed model is based on indicators with a value that is readily available, provides scores that vary among countries and over time, and in addition, reliably captures levels of severity. The developed heat maps visually illustrate the severity and the variation among countries. They show that both vulnerability and exposure together form the severity of a CE, while the scoring provides measurable comparisons that can support needs based decision-making.

The results in figure 7 show a high correlation between excess mortality and severity score, which indicates that the model is capable of capturing severity when using excess mortality as an outcome variable. Considerable inaccuracy may exist in available data on excess mortality,. The results from the assessment of reliability must therefore be interpreted with caution.

In the scoring of severity Somalia and the Central African Republic stood out as the most severe contexts during 2015, while the Syrian Republic and region only reached a moderate severity. This is in line with the estimated excess mortality as an absolute measurement of severity for historical CE. Here the indirect mortality due to a CE is much higher in countries with higher vulnerability, meaning that while the conflict in Syria in 2015 was, and still is, very violent with high numbers of direct victims of violence, the number of indirect deaths were likely lower than in Central African Republic and Somalia. As the number and proportion of people in need and displaced in Syria and the region is very high compared to any other ongoing CE, this makes the need score the highest in the assessment for 2015, regardless of the moderate severity.

he 7eed model with the suggested scoring levels captures variations of severity between CE affected countries in more detail than existing indices. As such, it may serve as a useful complement to measuring severity. However, it must be highlighted that the model compares “the worst of the worst” and a low severity score does not automatically imply a lack of need.

There are several limitations to the model and our assessment of the model. First, it is based on a limited number of vulnerability indicators that have been selected for their relevance and availability, in addition to ease of use. Priority has been given to indicators that have a quantifiable value. In some cases, despite being readily available, some of the vulnerability indicator values were several years old, which introduced imprecision. In contrast, indicators for exposure were frequently updated and presented, for example in the UN appeals. The validity of these data could be a concern. For instance, the definition for and methods to estimate people “in need”, as well as the previously used people “affected”, remain subjective and imprecise. We are also aware that in calculating the total need score, we are using the same indicator value twice - “people in need” - and that this is a potential source of error for the model. Nevertheless, in absence of more reliable data, this data remains the most useful.

In our analysis we relied on information from the institutions requesting funding, which could bring the independence of the analysis into question. The 7eed model is, however, a potential tool for a donor, where key information from the appeals can be interpreted and analysed among countries and across years. It is a way to make use of the available data in a systematic and objective manner that provides transparently derived results.

The 7eed model results must always be interpreted in context. They do not provide exact guidance on the absolute funding needs, but serve as a support tool together with other facts and considerations. Additional qualitative information must be part of the narrative analysis, such as violations of human rights and other factors that our model does not capture. The 7eed model also doesn’t take into account important regional or local variation, and is meant to be used at macro level.

While the scoring may provide information on the severity of a situation it does for instance not take into account so called “donor fatigue”, that may occur for contexts where the CE is lasting over longer periods, sometimes decades without any significant improvement of the situation or with a peaceful solution in sight, nor does it pay attention to geopolitical considerations that may influence funding decisions. More studies are therefore needed to document the significance of needs in relation to other factors that influence funding decision. The 7eed model is developed for ease of use and interpretation. It uses few indicators and a basic scoring system and where the information search and scoring requires a few minutes. It can be used for comparisons across countries. To what extent donors are ready implement such an analytical tool individually or per donor remains to be seen. The model could potentially also serve as a severity index or as a part of a severity index to be publicised and made available for donors and other stakeholders.

More than a decade has gone by with limited progress towards the goal of needs-based resource allocations. We believe that the results of this assessment are a promising step towards more systematic needs-based funding. We encourage other colleagues to assist in exploring humanitarian funding processes and assessment of needs-based allocations.

## Appendix 1

**Figure:**
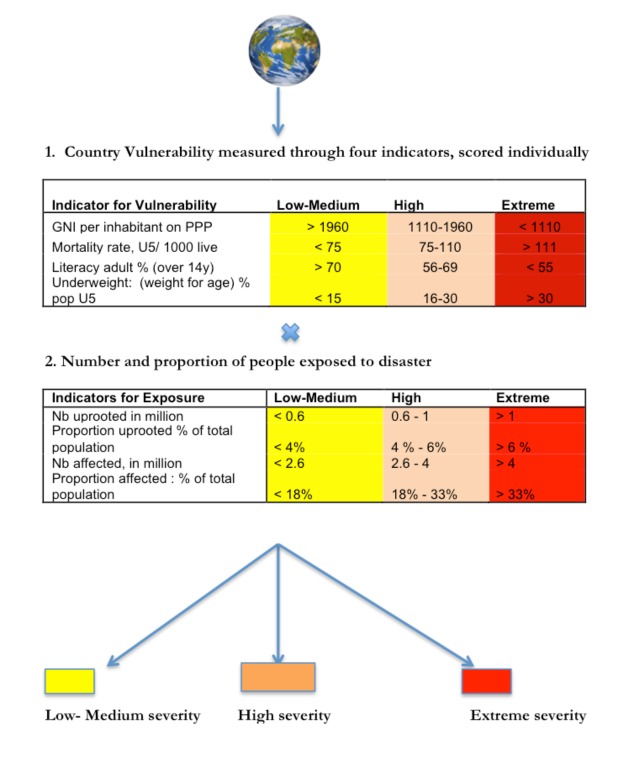
**The 7eed model**

## Competing Interests

The authors declare that they have no competing interests that have or may have influenced this study: the study design, data collection, data analysis, decision to publish, or preparation of the manuscript.
